# A comparative analysis of *SLA-DRB1* genetic diversity in Colombian (creoles and commercial line) and worldwide swine populations

**DOI:** 10.1038/s41598-021-83637-8

**Published:** 2021-02-22

**Authors:** Carmen Teresa Celis-Giraldo, Michel David Bohórquez, Milena Camargo, Carlos Fernando Suárez, Anny Camargo, Kewin Rodríguez-Obediente, Alejandra Martínez, Carlos Edmundo Lucero, Byron Hernández, Raúl Manzano-Román, Manuel Alfonso Patarroyo

**Affiliations:** 1 Animal Science Faculty, Universidad de Ciencias Aplicadas y Ambientales (U.D.C.A), 111166 Bogotá, Colombia; 2 PhD Programme in Biomedical and Biological Sciences, School of Medicine and Health Sciences, Universidad del Rosario, 112111 Bogotá, Colombia; 3 Molecular Biology and Immunology Department, Fundación Instituto de Inmunología de Colombia (FIDIC), 111321 Bogotá, Colombia; 4 Microbiology Postgraduate Programme, Universidad Nacional de Colombia, 111321 Bogotá, Colombia; 5 Germplasm Bank, Agrosavia, 250047 Bogotá, Colombia; 6 Proteomics Unit, Cancer Research Center (IBMCC/CSIC/USAL/IBSAL), 37007 Salamanca, Spain; 7 Faculty of Medicine, Universidad Nacional de Colombia, 111321 Bogotá, Colombia; 8 Health Sciences Division, Main Campus, Universidad Santo Tomás, 110231 Bogotá, Colombia

**Keywords:** Population genetics, DNA sequencing

## Abstract

Analysing pig class II mayor histocompatibility complex (MHC) molecules is mainly related to antigen presentation. Identifying frequently-occurring alleles in pig populations is an important aspect to be considered when developing peptide-based vaccines. Colombian creole pig populations have had to adapt to local conditions since entering Colombia; a recent census has shown low amounts of pigs which is why they are considered protected by the Colombian government. Commercial hybrids are more attractive regarding production. This research has been aimed at describing the allele distribution of Colombian pigs from diverse genetic backgrounds and comparing Colombian *SLA-DRB1* locus diversity to that of internationally reported populations. Twenty *SLA-DRB1* alleles were identified in the six populations analysed here using sequence-based typing. The amount of alleles ranged from six (Manta and Casco Mula) to nine (San Pedreño). Only one allele (01:02) having > 5% frequency was shared by all three commercial line populations. Allele 02:01:01 was shared by five populations (around > 5% frequency). Global F_ST_ indicated that pig populations were clearly structured, as 20.6% of total allele frequency variation was explained by differences between populations (*F*_*ST*_ = 0.206). This study’s results confirmed that the greatest diversity occurred in wild boars, thereby contrasting with low diversity in domestic pig populations.

## Introduction

Pig species are of great interest as a source of protein for human beings and due to their close relationship to humans in terms of anatomy, physiology and genetics^1–3^. This makes them a good model for biomedical research in areas such as immunology, therapeutics, production and xenotransplantation; characterising their immunological systems will lead to advances in such disciplines^4–6^. MHC antigen presentation has been widely studied in various species regarding functions associated with the immune system^7^. Genetic diversity has been comparatively analysed in the *Suidae* family concerning *Sus scrofa* (wild boars) and their congener *Sus scrofa domesticus* (domestic pig), along with independent analysis^8–12^.

The MHC is called the swine leukocyte antigen (SLA) in the *Sus scrofa* species; it is located on chromosome 7 and is formed by class I and class III (position 7p1.1) and class II complex regions (position 7q1.1)^10^. Class I and class II complex genes are related to immunity and inflammation and are characterised by being highly polymorphic^4^. Interestingly, the class II complex expresses proteins on the surface of antigen presenting cells (e.g. macrophages and dendritic cells); forming a protein complex, peptides anchored to the peptide-binding region (PBR) and the T-lymphocyte receptor (TCR) promote a humoral immune response^13–17^. *SLA-DRB1* (99 alleles) and *SLA-DQB1* (53 alleles) have the greatest polymorphism (https://www.ebi.ac.uk/ipd/mhc/). These genes consist of six exons; exon 2 encodes the PBR and has the greatest polymorphism^4,10,12^, thereby providing it with greater diversity for presenting peptides^18^. This study did not consider analysing the *SLA-DRA* gene because it has poor genetic variability, as happens in humans^4,10,19^.

Studies have evaluated MHC-Class II haplotype relationship with such parameters as production^20^, reproduction^21^ and/or genetic characterisation^8,13,22^. Several studies in the field of biomedical research have concluded that *SLA-DRB1* is an xenoantigen^5^; some studies have even been aimed at determining which pig alleles have the greatest reactivity^23^ whilst others have dealt with xenotransplantation^5,24,25^. MHC gene variability could be affected by natural and/or artificial selection^26,27^; regarding diversity, allele determination in a target population is thus relevant when designing epitope-based vaccines where it is recommended that the most prevalent pig population alleles (super-types) are identified so that such vaccines are designed to serve the majority^28,29^.

Local pigs are used in various traditional production systems worldwide, including native pigs in Asia, Africa and the Mediterranean (Iberian pigs); they are called creole pigs in Latin American countries^30^. The different breeds used in the modern pig industry have a greater impact on the rural economy because they are considered a low-cost protein source^31,32^. The appearance of the domestic pig in America arose from the introduction of Iberian or hairless pigs during the time of the Conquest; they have become adapted to local conditions over a period lasting more than 500 years, having some special phenotypical characteristics^31,33^. Three breeds are currently located in specific areas of Colombia. The nucleus of the Casco Mula breed is on Colombia’s Eastern Plains (Llanos Orientales) and the foothills of the Eastern Plains region (Piedemonte Llanero), the San Pedreño breed is in the Antioquia and Viejo Caldas departments and the Zungo breed is found on the Atlantic coast (mainly in the Córdoba department). The latter region is considered the place where the first specimens arrived in Colombia^34,35^.

Phenotypically, the San Pedreño breed is black, has a small head, a short snout and straight/upright ears; the Casco Mula breed’s name arises from syndactyly, as the pigs are multi-coloured, have red and sometimes black hair, a medium-sized snout, concave head forward-sloping, large ears and strong, short legs. The Zungo breed is characterised by being black and hairless, large-sized and having floppy ears; it has been reported that pigs from the Caribbean region have been crossed with pigs from the Duroc, Poland China and Hampshire breeds^31^.

These breeds’ living nuclei are kept in germplasm banks in the Colombian Agricultural Research Corporation’s (AGROSAVIA) research centres because they have a nucleus consisting of less than 1000 individuals^35^. According to previous reports these breeds are characterised by being able to produce meat and reproduce in difficult environmental situations^32^. Several studies have used genetic markers for better characterising breeds regarding their production potential^32–34,36^; a germplasm bank-based study of the San Pedreño breed recently characterised its genetic structure^35^.

The creole pig’s adaptation to varying environmental conditions has promoted the development of their productive and reproductive abilities. Production-related genetic improvement programmes in Colombia have enabled the development of commercial hybrids whose production-related qualities abilities have favoured their widespread distribution in Colombia (www.solla.com)^32,37^. This research was thus aimed at genotyping the *SLA-DRB1* locus in pig populations from Colombian creole breeds and commercial hybrids and comparing the diversity amongst Colombian populations and to that of some previously reported international ones.

## Results

### The study populations’ genetic diversity indices and phylogenetic analysis

**Figure 1:**
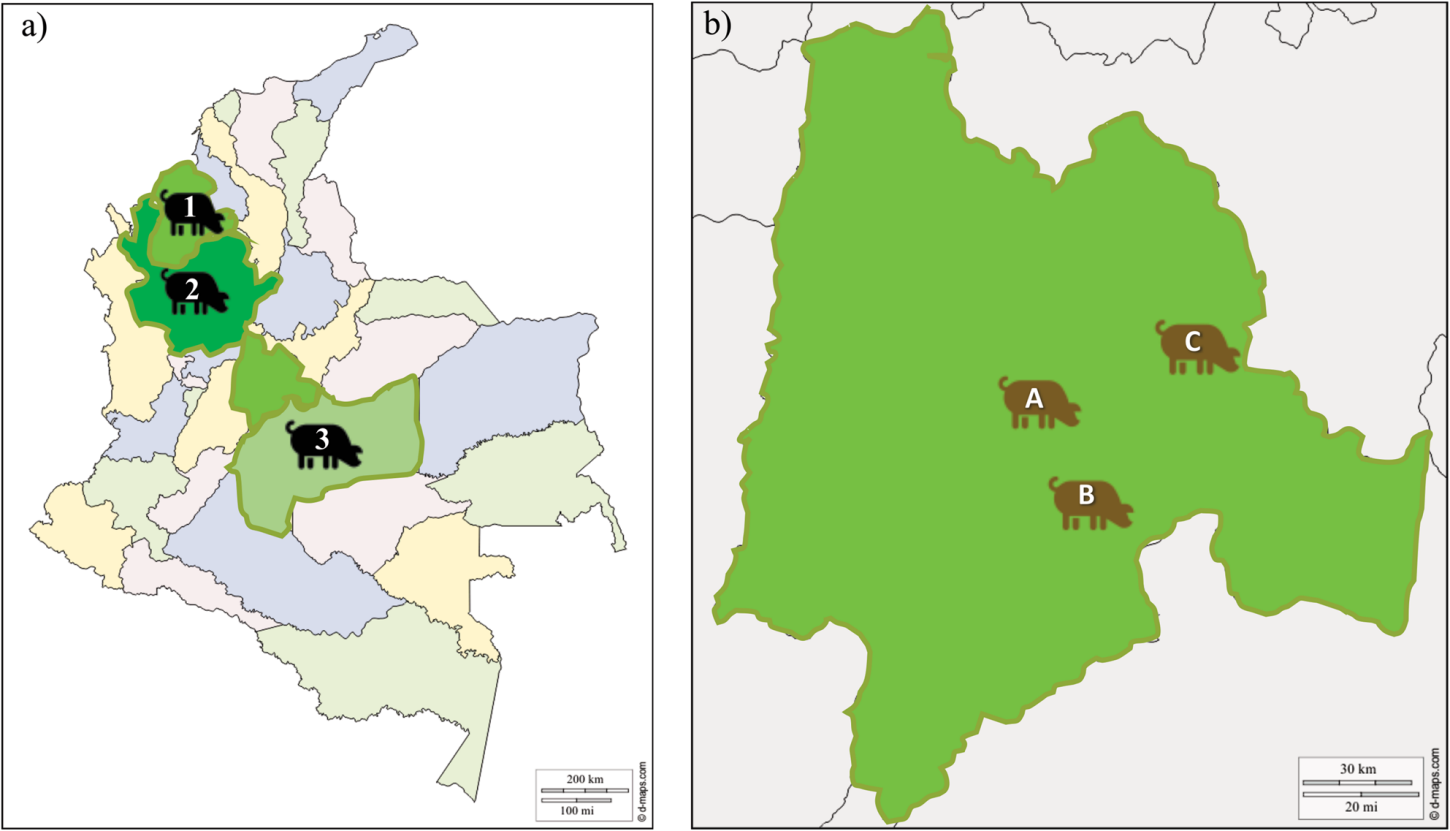
Sampling location sites. (**a**) Map of Colombia showing the location of AGROSAVIA’s research centres (I.C) having creole pig populations (pictogram in black), 1 = Zungo (IC Turipaná, Córdoba, n = 26), 2 = San Pedreño (IC El Nus, Antioquia, n = 35) and 3 = Casco Mula (IC La Libertad, Meta, n = 30)^82^. (**b**) Enlargement of the Cundinamarca department showing the location of the commercial line population (pictogram in brown), A = El Remanso-U.D.C.A (n = 31), B = Ubaque (n = 35) and C = Manta (n = 31). These free maps were downloaded from d-maps (https://d-maps.com/); the images are freely available and modifiable in accordance with d-maps policies^83,84^. Maps were modified using PowerPoint Software (Version 2002) Microsoft Office 365 ProPlus (U.D.C.A institutional license) by CTCG”.

The study involved sampling 188 pigs from locations in Colombia; 91 pigs were creoles and 97 came from a commercial line (Fig. 1). Twenty *SLA-DRB1* alleles were found in the population, five of which were only found in creole pigs (i.e. SLA-DRB1*02:05, *04:01, *09:01:01) and another seven were unique for the commercial pig group (i.e. SLA-DRB1*01:01, *02:11, *10:01:01).

**Figure 2:**
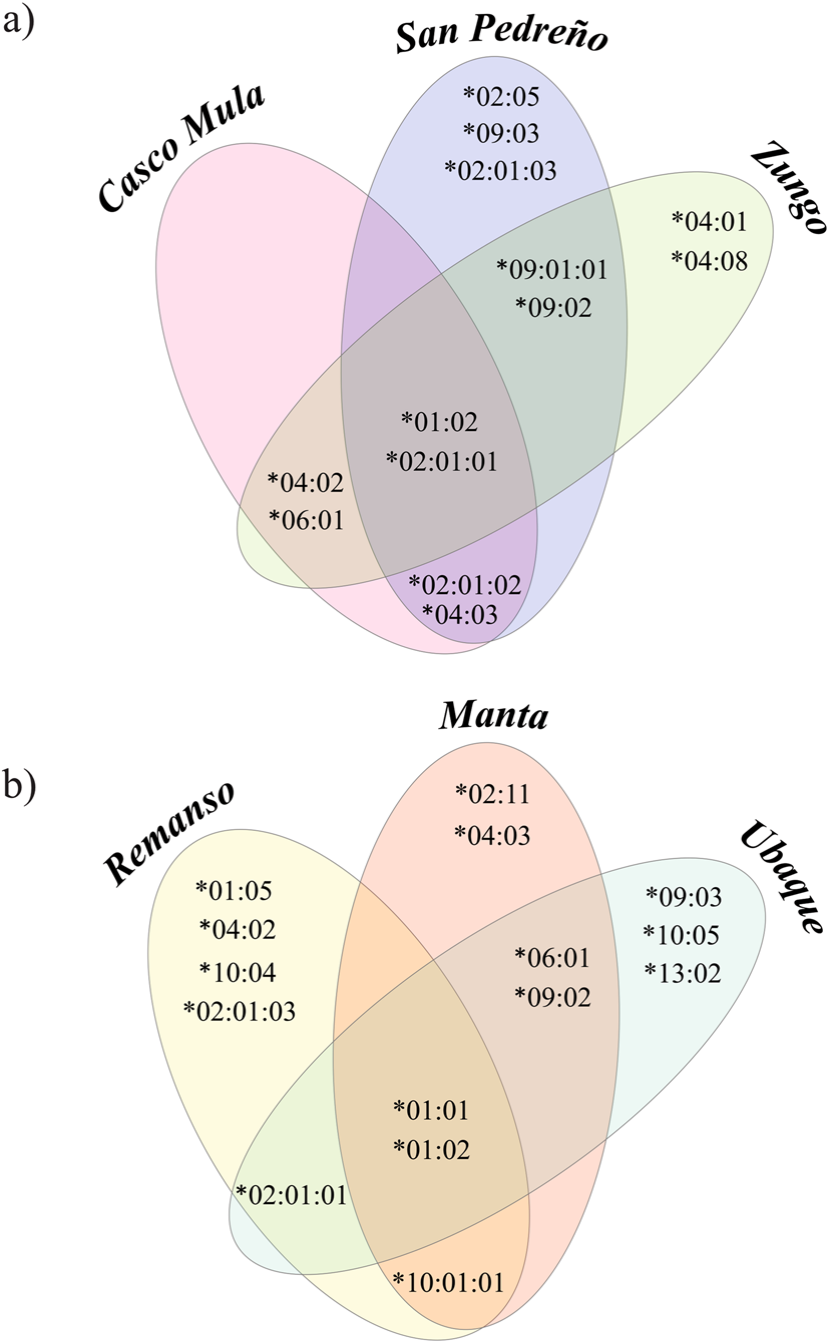
Venn diagram comparing the distribution of specific and shared *SLA-DRB1* alleles according to the target population. (**a**) Creole pigs. (**b**) commercial line.

Regarding allele distribution according to population, the creole pig results showed that Casco Mula, San Pedreño and Zungo shared eight alleles; two alleles (SLA-DRB1*01:02 and *02:01:01) being found in all three breeds. The SLA-DRB1*02:01:01 allele was shared by five populations where it occurred with > 5% frequency; moreover, this allele had > 40% frequency in creole populations. The San Pedreño population had the greatest amount of alleles (Fig. 2a, Table S1). Eight alleles were shared amongst individuals from the commercial line (Remanso, Manta and Ubaque) and two alleles (SLA-DRB1*01:01, *01:02) were shared amongst the three populations. Four unique alleles (SLA-DRB1*01:05, *02:01:03, *04:02 and *10:04) were observed in the Remanso population (U.D.C.A) (Fig. 2b).

**Figure 3:**
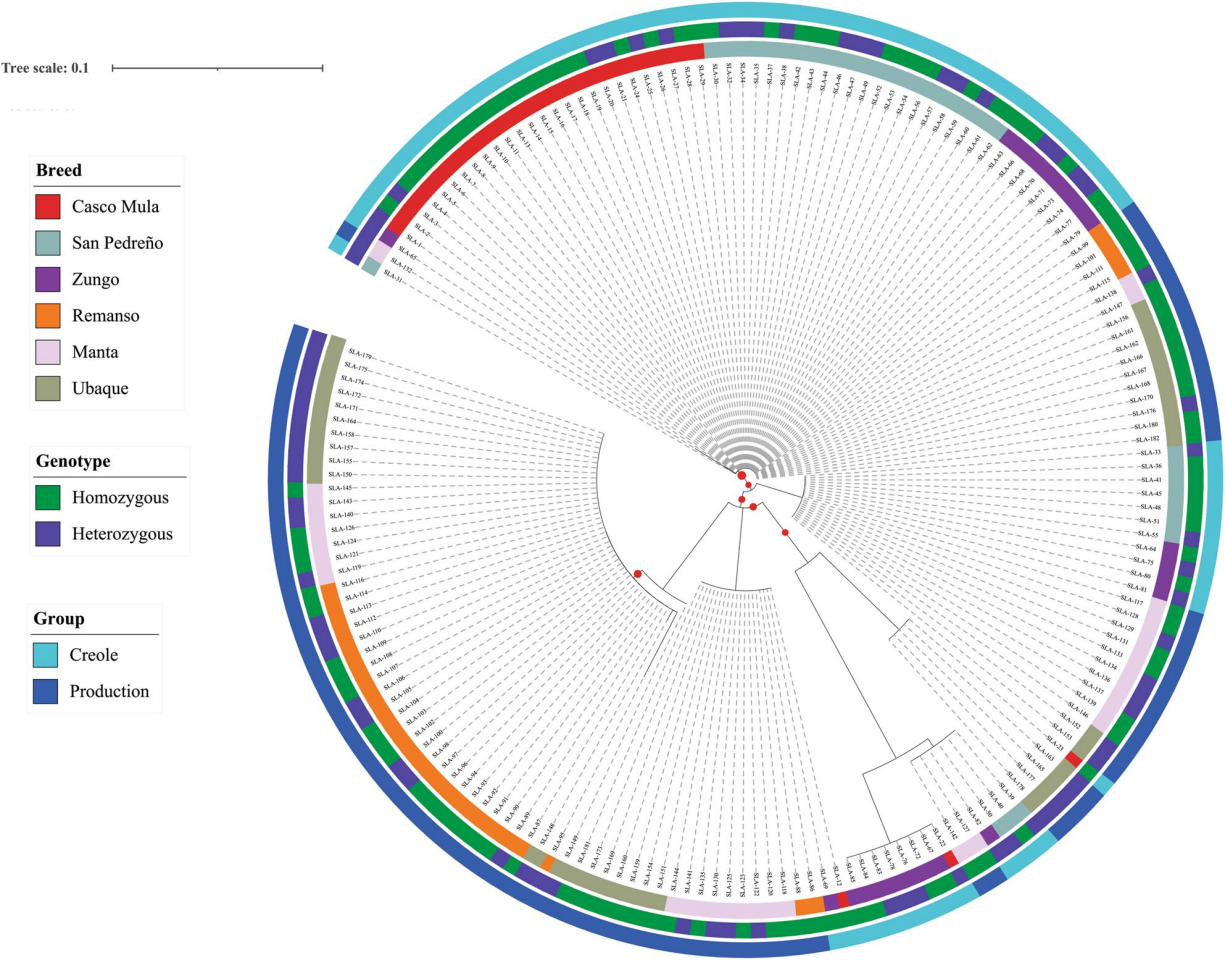
Homozygosity and heterozygosity distribution according to the populations analysed here.

Commercial line individuals had greater homozygosity (64%), the highest being observed in the Manta population (74.2%), followed by Remanso (71.0%). The Casco Mula population had the greatest homozygosity in the creole pig group (73.3%) (Fig. 3).

Diversity index results per population showed that the San Pedreño creole pig breed had the fewest segregating sites (S: 39) and haplotypes (h: 9), whilst the Zungo breed had the greatest nucleotide (π: 0.09214) and haplotype (Hd: 0.753) diversity (Table 1). The pig production group had the greatest nucleotide diversity (π: 0.06942), theta from eta (Θ: 0.06404) and haplotype diversity (Hd: 0.797) in the Manta population (Table 1).

**Table 1:** Genetic diversity indices for the breeds analysed in this study.

Parameter	Creole			Commercial line		
	Casco Mula	San Pedreño	Zungo	Remanso	Manta	Ubaque
*n*	60	70	52	62	62	70
π	0.04018	0.04262	0.09214	0.03761	0.06942	0.05807
Θ	0.05847	0.04102	0.06767	0.05394	0.06404	0.04934
S	64	39	68	47	66	54
h	6	9	8	8	7	8
Hd	0.491	0.593	0.753	0.567	0.797	0.788
SD	0.078	0.042	0.038	0.070	0.030	0.031

*n* the amount of sequences, *π* nucleotide diversity, *Θ* theta (per site) from eta, *S* the amount of segregating sites, *h* the amount of haplotypes, *Hd* haplotype diversity, *SD* standard deviation.

The amount of recombination events for creole pigs (3 events) was slightly higher than that for the production animals (2 events). No recombination events were found regarding Casco Mula; the recombinant SLA-DRB1*09:02 allele was found in both populations (Fig. S2).

### *SLA-DRB1* allele distribution compared to that in other populations

DRB1*02:01:01, *10:01:01, *01:01, *09:01:01 and *01:02 alleles were found (often having very high frequencies) in several Colombian, European and Asian populations. Several alleles were unique to one population and often found to have low frequency, including the DRB1*10:04 (Remanso) and *02:05 (San Pedreño) alleles. Some alleles were not found in Colombian populations, being restricted to just some European (DRB1*06:02, *06:03, *06:07, *05:01 and *13:01) or both European and Asian populations (DRB1*04:04, *08:01 and *14:01) (Table S3).

### Hardy–Weinberg equilibrium

Observed and expected heterozygosity and F_IS_ were determined for these populations (Table 2) to examine *SLA-DRB1* locus variability and potential Hardy–Weinberg variation. It was found that *ho* was lower in the commercial line than in creole pigs, the Zungo (*ho*:0.538) breed having the greatest *ho* amongst all six populations, whereas Casco Mula (*ho*:0.267) had a *ho* comparable to that of Remanso (*ho:*0.290) and Manta (*ho*:0.258). Casco Mula (*he*:0.380) had the lowest *he* value, which was twice as low as those for the San Pedreño (*he*:0.728) and Zungo (*he*:0.743) breeds. Remanso (*he*:0.558) and Manta (*he*:0.563) had the lowest *he* values amongst commercial line populations, due to the extremely biased allele frequency distribution in these populations. All populations had higher expected than observed heterozygosity and had significant heterozygote deficiency, being almost twice as high in the Manta (highest value) than the Casco Mula breed (lowest value). Both commercial line and creole pigs had the lowest observed heterozygosity (except for Duroc pigs) compared to that for other pig populations, although expected heterozygosity levels were more similar to those for other populations. Allele richness (the amount of alleles corrected for sampling size effects) had a clear sample size-related effect on the amount of alleles identified (Table 2). As expected, neither *ho* nor *he* appeared to be affected by sample size (*ho* r = 0.13 and *he* r = 0.17), indicating that these estimates should be good representatives for *SLA-DRB1* diversity in these pig populations. Colombian creole and commercial line pig populations had the highest Hardy–Weinberg variations (highest *F*_*IS*_ values).

**Table 2:** Sample size (N), amount of alleles (Na), allele richness (*Rs*), observed heterozygosity (*ho*), expected heterozygosity (*he*) and *F*_*IS*_ values for analysed pig populations.

Population	N	N_a_	*R*_*s*_	*h*_*o*_	*h*_*e*_	*F*_*IS*_ (S.E.)—*p*-value
**Remanso**	31	8	5.816	0.290	0.558	0.4842 (0.0011)—0.0011
**Manta**	31	7	5.478	0.258	0.563	0.5463 (0.0000)—< 0.0001
**Ubaque**	35	8	5.478	0.514	0.731	0.2994 (0.0034)—0.0101
**Casco Mula**	30	6	4.542	0.267	0.380	0.3401 (0.0033)—0.0203
**San Pedreño**	35	9	7.128	0.457	0.728	0.3885 (0.0000)—< 0.0001
**Zungo**	26	8	6.566	0.538	0.743	0.3739 (0.0064)—0.0238
Wild boar	125	19	10.646	0.720	0.885	0.1879 (0.0000)—< 0.0001
Micromini	14	8	8.000	0.785	0.867	0.0978 (0.0150)—0.6968
SNU	114	4	3.993	0.657	0.715	0.0813 (0.0009)—0.0046
Yorkshire	31	12	9.792	0.741	0.878	0.1575 (0.0018)—0.0046
Berkshire	31	8	6.614	0.677	0.760	0.1108 (0.0120)—0.1202
KNP	105	6	4.939	0.771	0.744	− 0.0368 (0.0145)—0.4926
Duroc	32	6	4.774	0,281	0,489	0.0986 (0.0152)—0.0018
Landrace	30	9	8.141	0.766	0.849	0.0986 (0.0138)—0.1454
Landrace_C	22	5	4.869	0.681	0.734	0.0735 (0.0056)—0.1069
Yorkshire_C	15	6	5.867	0.866	0.721	− 0.2093 (0.0133)—0.3668
Pietrain	26	10	8.712	1.00	0.862	− 0.1638 (0.0205)—0.3823

*p* < 0.05.

The mean amount of nonsynonymous *(N*_*d*_*)* and synonymous *(S*_*d*_*)* polymorphisms was calculated to evaluate sequence diversity amongst populations. Considering the whole β1 domain, *S*_*d*_ ranged from 2.32 (Remanso) to 8.65 (Micromini) and *N*_*d*_ from 7.80 (Casco Mula) to 19.02 (KNP). Colombian populations had the lowest values for both *N*_*d*_ and *S*_*d*_, despite the amount of alleles or sample size being the lowest amongst all populations. This indicated a less divergent gene pool for the Colombian populations. When only considering PBR codons, *Sd* ranged from 0.70 (Remanso) to 2.79 (Micromini) and *N*_*d*_ from 7.0 (Duroc) to 11.11 (KNP); Colombian populations, Yorkshire_E, SNU and Duroc also had low levels of diversity. As expected, a large percentage (54% to 82%) of *N*_*d*_ in the β1 domain was due to PBR positions, confirming these positions being the most variable ones (Table 3).

**Table 3:** The amount of nonsynonymous (*N*_*d*_) and synonymous (*S*_*d*_) polymorphisms in the sequence encoding the whole β1 domain and peptide binding region (PBR) for the swine populations analysed here.

Population	β1 domain		Peptide binding region	
	*N*_*d*_ (SE)	*S*_*d*_ (SE)	*N*_*d*_ (SE)	*S*_*d*_ (SE)
**Remanso**	9.07 (1.94)	2.32 (0.75)	6.19 (1.38)	0.70 (0.34)
**Manta**	8.43 (1.60)	2.55 (0.58)	5.85 (1.13)	0.89 (0.38)
**Ubaque**	10.13 (2.29)	2.72 (0.94)	8.26 (1.77)	1.30 (0.66)
**Casco Mula**	7.80 (1.43)	2.61 (0.65)	5.17 (0.92)	0.82 (0.37)
**San Pedreño**	9.12 (2.06)	3.47 (0.86)	7.20 (1.55)	1.31 (0.56)
**Zungo**	15.95 (2.97)	8.07 (1.64)	10.52 (1.89)	2.49 (0.86)
Wild boar	17.34 (2.69)	7.63 (1.51)	10.44 (1.92)	2.52 (0.75)
Micromini	18.71 (3.00)	8.65 (1.59)	11.01 (1.69)	2.79 (0.90)
SNU	12.60 (2.58)	6.74 (1.50)	8.28 (1.81)	2.05 (0.79)
Yorkshire	17.98 (2.85)	6.80 (1.22)	11.09 (1.93)	1.81 (0.56)
Berkshire	13.91 (2.45)	3.87 (1.00)	9.48 (1.63)	1.12 (0.51)
KNP	19.02 (2.88)	7.67 (1.57)	11.11 (1.79)	1.90 (0.70)
Duroc	10.06 (1.73)	4.92 (0.91)	7.00 (1.12)	1.56 (0.44)
Landrace	17.54 (2.82)	5.09 (1.15)	10.56 (1.81)	1.24 (0.48)
Landrace_C	16.96 (2.73)	5.70 (1.31)	9.15 (1.70)	1.88 (0.60)
Yorkshire_C	12.10 (2.24)	4.58 (1.08)	7.59 (1.59)	1.18 (0.50)
Pietrain	18.24 (2.98)	7.26 (1.48)	10.61 (1.92)	1.95 (0.73)

*SE* standard error.

### Population structure and genetic differentiation

**Figure 4:**
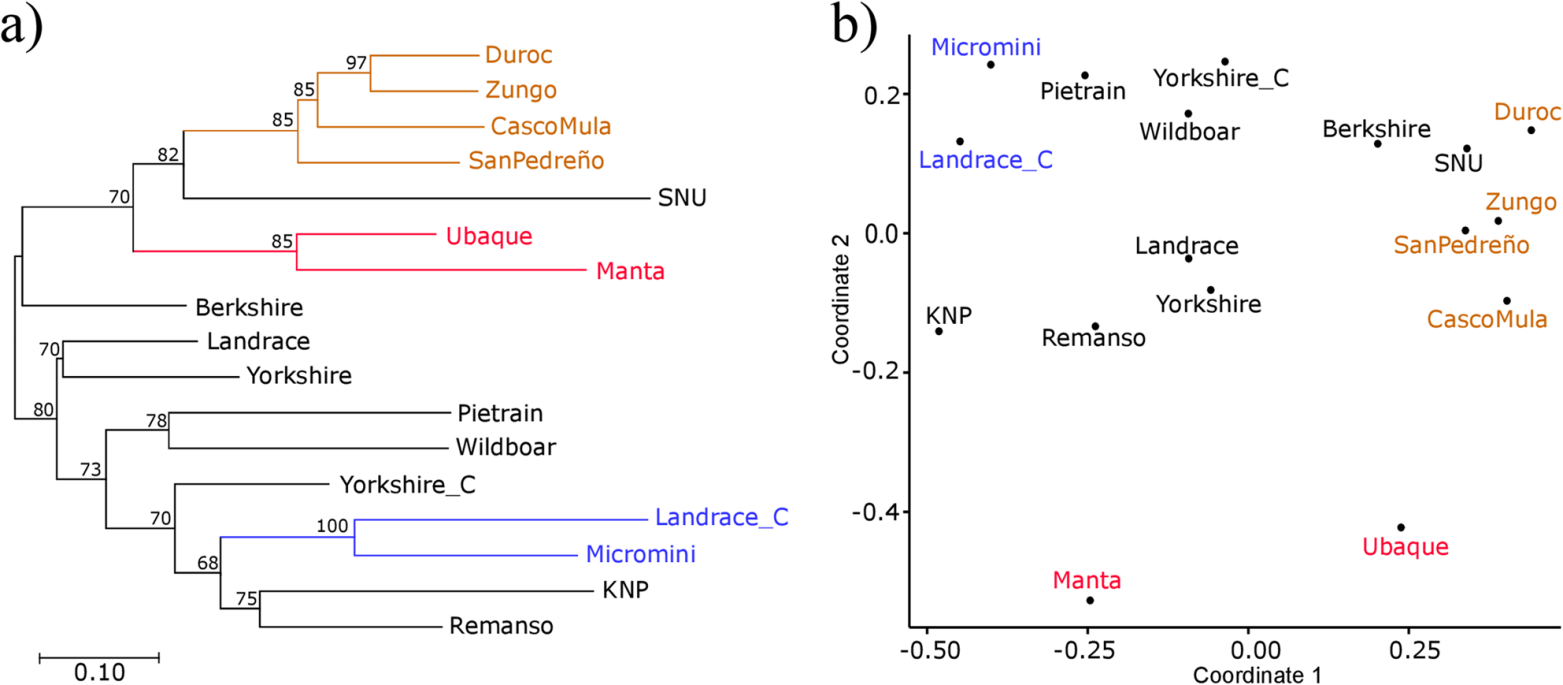
Relationships between pig populations. BIONJ dendrogram (**a**) and multidimensional scaling analysis (**b**) based on DA genetic distances calculated from *SLA-DRB1* allele frequency. Bootstrap values are shown for each node in the dendrogram and clusters identified by both methods are depicted in the same colour.

The overall F_ST_ indicated that the pig populations were clearly structured; 17.4% of total variation for allele frequency could be explained by differences between populations (*F*_*ST*_ = 0.174; *p*-value < 0.0001) (Table S4). D_A_ genetic distances based on allele frequency were used to construct dendrograms using the NJ and BIONJ algorithms to further evaluate relationships between populations, the BIONJ algorithm (Fig. 4a) having the best adequacy (*BIONJ RQS* = 0.698, *NJ RQS* = 0.667). Multidimensional scaling (MDS), using the D_A_ genetic distance matrix, was explored to depict population relationships in two dimensions (Fig. 4b). MDS also gave good representation of real variability (RQS = 0.663) and the results largely agreed with the BIONJ dendrogram.

Despite the populations’ homogeneous MDS distribution, three well-supported clusters could be identified in the dendrogram (bootstrap support ≥ 85). The first was a compact group consisting of Duroc, Zungo, Casco Mula and San Pedreño. This group had a 0.26 mean *D*_*A*_ distance and the alleles shared by its members (DRB1*01:02, *02:01:01, *02:01:02, *04:02, *04:03, *04:08, *06:01 and *09:01:01) accounted for 76% of its mean absolute allele frequency (Table 2). The second cluster was made up of Ubaque and Manta; it had a 0.358 *D*_*A*_ distance and 75% mean absolute frequency (explained by alleles DRB1*01:01, *01:02, *06:01 and *09:02). The third cluster consisted of Landrace_C and Micromini, having a 0.432 *D*_*A*_ distance and its common alleles (DRB1*06:01, *08:01 and *10:01:01) explained 63% of mean absolute allele frequency (Table 2). Yorkshire and Landrace clustered in both the MDS and the dendrogram and, although not very well supported by bootstrap, this cluster had a 0.259 *D*_*A*_ distance and 88% mean absolute allele frequency (alleles DRB1*01:01, *01:02, *02:01:01, *04:02, *04:04, *06:03, *09:01:01 and *10:01:01) (Fig. 4 and Table 2).

**Figure 5:**
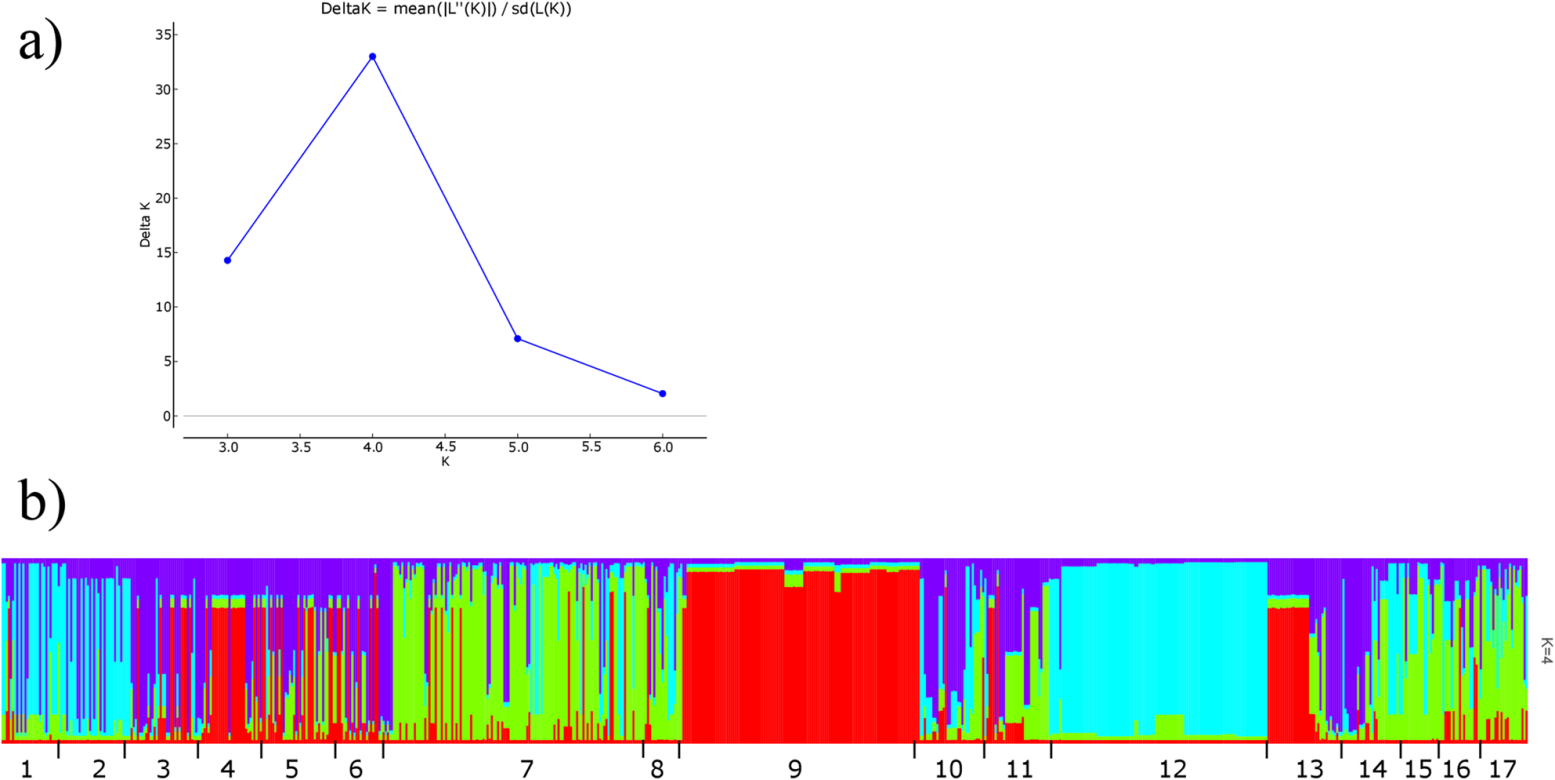
Pig population structure analysis based on Bayesian clustering. Delta K values as a function of K (**a**). Population clustering (**b**). Each vertical line represents an individual and each colour an inferred population. Numbers under (**b**) indicate populations: 1, Remanso; 2, Manta; 3, Ubaque; 4, Casco Mula; 5, San Pedreño; 6, Zungo; 7, Wild boar; 8, Micromini; 9, SNU; 10, Yorkshire; 11, Berkshire; 12, KNP; 13, Duroc; 14, Landrace; 15, Landrace_C; 16, Yorkshire_C; 17, Pietrain.

A clustering method was used for assigning individuals to populations according to their genotypes. Based on *ΔK* distribution, an inferred *K* value of 4 provided the best fit to the data and thus all individuals could be grouped in 4 populations (Fig. S5). Interestingly, KNP and SNU were well-defined populations having fewer admixed genotypes. Colombian populations could be separated into two groups. Creole pigs were admixed with SNU, agreeing with dendrogram and MDS ordination plots. Remanso and Manta genotypes were admixed with KNP, agreeing with first MSD coordinate (Fig. 5).

## Discussion

This study was aimed at comparing *SLA-DRB1* (Exon 2) genetic diversity for the first time in two Colombian pig populations. It was seen that the amount of alleles was similar in each population; the Casco Mula breed had the least amount of alleles. Individuals from the San Pedreño breed had the most alleles and individuals from the Zungo breed the highest nucleotide variability (Table 1). Such data coincided with that reported by other groups^26,38^, though differing from that reported by some authors^8,26^; for example, twenty alleles have been identified in a population of 133 wild boars in Croatia^8^. Sequence diversity analysis showed that most polymorphisms in the β1 domain were non-synonymous, Colombian pig populations having lower values in this region. However, synonymous and non-synonymous polymorphism distribution in the PBR was more uniform, despite the Colombian populations having less diversity. An excess of non-synonymous polymorphisms was evident in the PBR; this was to be expected due to this region’s role in antigen presentation^11,12,26^.

This study’s results confirmed greater diversity in wild boars, contrasting with low diversity in domestic pig populations. The clearest differences were observed regarding allele richness which was especially low in some populations (*R*_*s*_ < 5 in the Casco Mula, KNP, Duroc and Landrace_C populations). However, some populations having low *R*_*s*_ had high *h*_*e*_ (corrected for sample size), indicating a marked variation regarding allele frequency distribution.

Considering that genetic drift and the structure of animal crosses are factors affecting allele frequency distribution (having greater intensity in populations having smaller effective population size)^39–43^, then variations regarding expected heterozygosity and allele richness could have been due to the combined effects of small effective population sizes and varying degrees of endogamy. The latter factor would have mainly been due to the artificial selection of production features. For example, selection based on the Casco Mula breed’s characteristic feature (hoof) and its small effective population size^35^ could lead to intensive in-breeding which would then produce differences regarding allele frequency and could eventually lead to a loss of some alleles. This idea could also be supported by the high *F*_*IS*_ values for some of these populations, indicating higher homozygote percentages than those to be expected in a population in equilibrium and could suggest higher endogamy levels.

Alleles DRB1*01:02 and *10:01 were previously considered ancestral due to their presence in populations having different origins^26^. It was seen in this study that alleles DRB1*01:01, *01:02, *02:01:01, *09:01:01 and *10:01:01 were broadly distributed, often having high frequency in various populations. As the pigs’ domestication seems to have been occurred during six independent events^44^, these alleles’ distribution could either indicate their presence in the founding populations in only some domestication events or their presence in the founding populations in all domestication events, accompanied by the subsequent loss of some alleles due to drift and/or natural selection. Allele DRB1*02:01:01 may thus have been present in pig populations originating the creole and commercial line populations but which could have disappeared due to genetic drift (or its frequency could have become reduced and not been sampled) in the Manta population. In fact, several studies have established that mutationally robust populations might have less mutational load; however, it is not clear whether small populations have less susceptibility to drift-related fitness reduction^45^.

The creole pig group’s results particularly seem to have coincided with previously reported data regarding individuals from the San Pedreño breed^35^, confirmed by analysing pedigree data from information collected from 2008 to 2017 for estimating the generation interval, the level of pedigree integrity, consanguinity and evolution. It was particularly interesting that this group had 6.73% consanguinity^35^. There have been no reports concerning this for Casco Mula pigs to date.

Considering the group’s experience^46^, it was decided to use bioinformatics methods, involving Haplofinder software, for assigning alleles in heterozygous individuals; this tool has been used regarding bovine species for *DR*^47,48^ and *DQA*^49^, thereby facilitating assigning a large amount of individuals, resorting to cloning in just 16% of the population. The data obtained from analysing variability between populations revealed a low level of heterozygotes, this being more evident in individuals from the Casco Mula breed and those from the Manta and Remanso groups.

The recombination events observed in this study coincided with that reported by other authors^8,50^ and have been considered a molecular mechanism associated with MHC genetic diversity; such phenomenon has been observed in ungulate^51^ and passerine species^52^.

Studies in Poland regarding commercial line pigs and native individuals have analysed runs of homozygosity (ROH), identifying genome regions having high ROH levels, for example, SSC4 (51.9–55.9 Mb) which is related to various functions, including immunological ones^53^. Detecting high levels of homozygosity in various regions of the genome of inbred Babraham pigs led to developing specific MHC-related analysis, revealing a high level of homozygosity, thereby making it a candidate for research group use as a biomodel^54^.

The pig populations had a clear genetic structure based on the *SLA-DRB1* locus. Interestingly, the low genetic differentiation in Colombian creole populations contrasted sharply with the great differentiation between commercial line populations. This could have arisen from crossing individuals for obtaining such lines. Commercial lines’ impact on Landrace pigs’ genetic diversity has been evaluated recently; the current breed provides evidence of the old lines’ formation, but that such fusion has reduced Landrace genetic diversity^55^. Various clusters were identified based on a small genetic distance and/or high bootstrap support values. This indicated that the populations forming these clusters have high genetic identity and detailed analysis has revealed that they share a large percentage of their mean absolute allele frequency and the alleles so involved. Such information could be used in infectious disease control programmes or when designing vaccines^56–58^. The SLA-DRB1*02:01:01 allele has 79% amino acid identity with HLA-DRB1*01:01 in pigs and has been used for making a bioinformatics predictor based on pocket profile methodology^28^, highlighting the need for characterising SLA diversity when designing vaccination and control programmes.

## Materials and methods

### Animals and DNA isolation

The study involved sampling 188 animals from six populations, thereby identifying a first group called the creole pig group consisting of individuals from the Zungo (n = 26), Casco de Mula (n = 30) and San Pedreño populations (n = 35). All blood samples from this group were provided by AGROSAVIA’s research centres. A second group called the commercial line consisted of Super Mom 52 and PIC commercial hybrid populations; blood samples were taken on three pig farms in the Cundinamarca department, identified as follows: Remanso U.D.C.A (n = 31), Manta (n = 31) and Ubaque (n = 35) (Fig. 1). All the pigs were randomly sampled; variables such as age, gender, production stage or relationship were not considered.

Venous blood was obtained by jugular vein puncture and kept in tubes with anticoagulant (EDTA). Genomic material was extracted following Wizard Genomic DNA Purification Kit recommendations. All samples’ DNA integrity was determined on 1.5% agarose gel; a µDrop microplate reader was used for determining DNA concentration. The samples were then stored at − 20 °C until PCR. The sample collection procedure was approved by the Universidad de Ciencias Aplicadas y Ambientales’ (U.D.C.A) bioethics committee (in minutes No.002/2019). All procedures followed the guidelines and regulations established in the Colombian code of bioethics (resolution 8430 of 1993) and Law 84/1989 regarding the protection of animals.

Genotypical data regarding the populations reported in the pertinent literature was included for carrying out the comparative analysis of diversity between Colombian and international populations, such as Wild boar^8^, Microminipig^59^, SNU (Seoul National University), Yorkshire, Berkshire, Korean Native Pigs (KNP), Duroc and Landrace^14^, Landrace_C and Yorkshire_C^26^ and Pietrain pigs^6^.

### *SLA-DRB1* genotyping

Polymerase chain reaction (PCR) was used for obtaining the *SLA-DRB1* exon 2 sequences, using a pair of primers reported in the literature: forward 5′-GTCCACGCAGCGCATTTCTT-3′^38^ and reverse 3′-ACACACACTCTGCCCCCCG-5′^26^. Amplified fragment length was 328 bp; 2X high-fidelity Kodaq PCR MasterMix was used and reaction volume was 25 µL, distributed as follows: 7 µL nuclease-free water, 12.5 µL enzyme Master Mix, 1.25 µL for each primer [10 µM] and 3 µL genomic DNA (concentration ranged from 50–100 μg/µL). Amplification conditions were as follows: initial denaturing at 94 °C for 3 min, 35 cycles consisting of denaturing at 94 °C for 30 s., annealing at 68.2 °C for 30 s., extension at 72 °C for 1 min, followed by a final extension step at 72° for 5 min. All PCR products were amplified in 2 independent reactions, revealed on 2% agarose gel and then purified and sent to Macrogen Inc_._ (South Korea) to be sequenced in both senses.

CLC Main Workbench v.3.6.5 software (QIAGEN, Aarhus, Denmark; https://digitalinsights.qiagen.com) was used for assembling and analysing the sequences. Heterozygotic individuals’ sequences were manually edited, IUPAC/IUBMB single-letter code was used for ambiguous pairs^60^ and Haplofinder Python script was used for assigning them^47–49^. This involved creating a library with the exon 2 sequences from the alleles reported for *SLA-DRB1* in IPD-MHC (www.ebi.ac.uk/ipd/mhc). pGEM-T Easy vector (Promega) was used for ligating heterozygotic samples which could not be assigned by bioinformatics methods which were then transformed in competent *E. coli* JM109 cells to separate both alleles, following the protocol reported by FIDIC^61^. Selection involved blue/white colony screening; the amount of positive colonies was replicated and scaled for plasmid purification; 5–10 positive clones were obtained per sample, confirmed on 1.5% agarose gel and sent to Macrogen again to be sequenced in both senses. The complete genotyping results are shown in Table S6.

### Genetic diversity indices, recombination events and phylogenetic analysis

The MUSCLE sequence alignment tool (https://www.ebi.ac.uk/Tools/msa/muscle/) was used for aligning the sequences; they were then evaluated for identifying indels (insertion and elimination events) which could have affected the length of any sequence to be analysed. Minor adjustments were then made to the alignments to eliminate gaps. DnaSP (v5) software (http://www.ub.edu/dnasp/) was used for evaluating each population’s genetic diversity, nucleotide diversity (π), theta (per site) from eta (Θ), the amount of segregating sites (S), the amount of haplotypes (h) and haplotype diversity (Hd) along with their standard deviations (SD).

Maximum likelihood trees with double precision, considering Jukes-Cantor as a substitution model were built based on the alignments using FastTree Version 2.1.9 (http://www.microbesonline.org/fasttree/) for phylogenetic reconstruction; node robustness was evaluated by bootstrap method with 1000 replicates^62^. The phylogenetic trees were visualised in the web tool Interactive Tree Of Life V3 (http://itol.embl.de)^63^; the Recombination Detection Program (version 4) (RDP4) was also used. In-depth statistical analysis included using RDP, GENECONV (for detecting gene conversion), BootScan (screening nucleotide sequence alignments for evidence of recombination ), Maximum Chi-Squared test (MaxiChi) (for identifying potential recombination events between two sequences or between two sequences and a putative derived sequence), Chimaera (for identifying recombinant and parental sequences and estimate breakpoint positions), Sister Scanning (SiScan) (for assessing phylogenetic and compositional signals in various patterns of identity), 3Seq (for ascertaining whether any sequence in a data set is a recombinant or mosaic), VisRD (for visual recombination detection in a sequence alignment) and BURT (for testing significance in factor analysis/analysis of variance) methods^64^.

### Measures of genetic diversity and Hardy–Weinberg equilibrium

The maximum likelihood method was used for obtaining the amount of alleles (Na) and allele frequencies by direct counting, along with their standard errors^65^. FSTAT software was used for calculating allele richness^66^. Observed heterozygosity (*ho*) and unbiased expected heterozygosity (*he*) according to Nei^67^ were estimated using Arlequin v.3.5 software for population genetic analysis^68^. Potential Hardy–Weinberg variation percentages were estimated by *F*_*IS*_ statistic test^69^ included in Genepop v.4.7.0, using the exact test of significance^70^. Molecular Evolutionary Genetics Analysis (MEGA X)^71^ software was used for calculating the average amount of nonsynonymous (*N*_*d*_) and synonymous (*S*_*d*_) polymorphisms and their standard errors (by bootstrapping with 10,000 replicates) for all sequence pairs within populations. *N*_*d*_ and *S*_*d*_ were not normalised for sequence length to attain the percentage of polymorphisms in the PBR regarding the whole β1 domain encoding sequence.

### Population structure and genetic differentiation

Arlequin software v.3.5.2 analysis of molecular variance (AMOVA)^68,72^ was used for assessing variation amongst populations. POPTREE2 software^73^ was used for estimating D_A_ genetic distances between populations from allele frequencies^74^. R packages *ape *^75^ and *poppr*^76^ were used to estimate Nei standard genetic distances between populations from amino acid sequences^77^. R packages *ape*, *poppr* and *stats* were used for inferring dendrograms with NJ and BIONJ algorithms. Confidence values for nodes in the dendrogram were obtained by bootstrapping alleles with 1,000 replicates and the trees were constructed using webserver Phylogeny.fr^78^. Multidimensional Scaling (MDS) analysis based on D_A_ genetic distances involved using the R *cmdscale* function. R squared was used for evaluating the representation of the trees’ original distances and MDS and correlation analysis was used for evaluating sample size effects on *h*_*e*_ and *h*_*o*_. The Bayesian iterative algorithm in STRUCTURE multilocus genotype data for assigning individuals to a particular population^79^ was used for further evaluation of population structure, inferring genetic clusters in which individuals could be assigned. Structure harvester^80^ a programme for parsing Pritchard’s STRUCTURE output and performing the Evanno method^81^ was used for determining the most likely *K* by *ΔK* distribution. Ten replicates were run for each *K* using the admixture model with correlated allele frequencies and Markov Chain Monte Carlo 50,000 burn-in steps, followed by 100,000 iterations.

## Supplementary Information

Supplementary Information
