# Supplementary material for: A comparative analysis of SLA-DRB1 genetic diversity in Colombian (creoles and commercial line) and worldwide swine populations
Source: Sci Rep. 2021 Feb 22;11:4340. doi: 10.1038/s41598-021-83637-8 (PMC7900169; doi:10.1038/s41598-021-83637-8)
Supplement: Supplementary file 1 — Supplementary Information [file 41598_2021_83637_MOESM1_ESM.pdf]

Supplementary information

A comparative analysis of *SLA-DRB1* genetic diversity in Colombian (creoles and commercial line) and worldwide swine populations

Carmen Teresa Celis-Giraldo[1,2¶], Michel David Bohórquez[3,4¶], Milena Camargo[2,3¶], Carlos Fernando Suárez[3], Anny Camargo[2,3], Kewin Rodríguez-Obediente[3,4], Alejandra Martínez[1], Carlos Edmundo Lucero[5], Byron Hernández[5], Raúl Manzano-Román[6], Manuel Alfonso Patarroyo[3,7,8*]

[1] Animal Science Faculty, Universidad de Ciencias Aplicadas y Ambientales (U.D.C.A.), 111166 Bogotá, Colombia

[2] PhD Programme in Biomedical and Biological Sciences, School of Medicine and Health Sciences, Universidad del Rosario, 112111 Bogotá, Colombia

[3] Molecular Biology and Immunology Department, Fundación Instituto de Inmunología de Colombia (FIDIC), 111321 Bogotá, Colombia

[4] Microbiology Postgraduate Programme, Universidad Nacional de Colombia, 111321 Bogotá, Colombia

[5] Germplasm Bank, Agrosavia, 250047 Bogotá, Colombia

[6] Proteomics Unit, Cancer Research Centre (IBMCC/CSIC/USAL/IBSAL), 37007 Salamanca, Spain

[7] Faculty of Medicine, Universidad Nacional de Colombia, 111321 Bogotá, Colombia

[8] Health Sciences Division, Main Campus, Universidad Santo Tomás, 110231 Bogotá, Colombia

[*] Corresponding author
e-mail: mapatarr.fidic@gmail.com

[¶] These authors contributed equally to this work.

Table S1. Allele frequency and related SE.

Allele	Remanso	Manta	Ubaque	Casco Mula	San Pedreño	Zungo
*01:01	0.048 ± 0.027	0.645 ± 0.06	0.2 ± 0.047	0	0	0
*01:02	0.161 ± 0.046	0.112 ± 0.04	0.328 ± 0.056	0.066 ± 0.032	0.028 ± 0.02	0.038 ± 0.028
*01:05	0.016 ± 0.015	0	0	0	0	0
*02:01:01	0.064 ± 0.031	0	0.357 ± 0.057	0.783 ± 0.054	0.471 ± 0.061	0.403 ± 0.072
*02:01:02	0	0	0	0.016 ± 0.016	0.057 ± 0.028	0
*02:01:03	0.032 ± 0.022	0	0	0	0.028 ± 0.02	0
*02:05	0	0	0	0	0.014 ± 0.014	0
*02:11	0	0.016 ± 0.015	0	0	0	0
*04:01	0	0	0	0	0	0.019 ± 0.02
*04:02	0.016 ± 0.015	0	0	0.033 ± 0.023	0	0.038 ± 0.028
*04:03	0	0.064 ± 0.031	0	0.016 ± 0.016	0.085 ± 0.034	0
*04:08	0	0	0	0	0	0.288 ± 0.066
*06:01	0	0.048 ± 0.027	0.057 ± 0.027	0.083 ± 0.036	0	0.019 ± 0.02
*09:01:01	0	0	0	0	0.2 ± 0.049	0.134 ± 0.05
*09:02	0	0.096 ± 0.037	0.014 ± 0.014	0	0.085 ± 0.034	0.057 ± 0.034
*09:03	0	0	0.014 ± 0.014	0	0.028 ± 0.02	0
*10:01:01	0.645 ± 0.06	0.016 ± 0.015	0	0	0	0
*10:04	0.016 ± 0.015	0	0	0	0	0
*10:05	0	0	0.014 ± 0.014	0	0	0
*13:02	0	0	0.014 ± 0.014	0	0	0

* Maximum likelihood estimates of allele frequency and their standard errors (SE)

Fig S2. Recombination events in the target population.

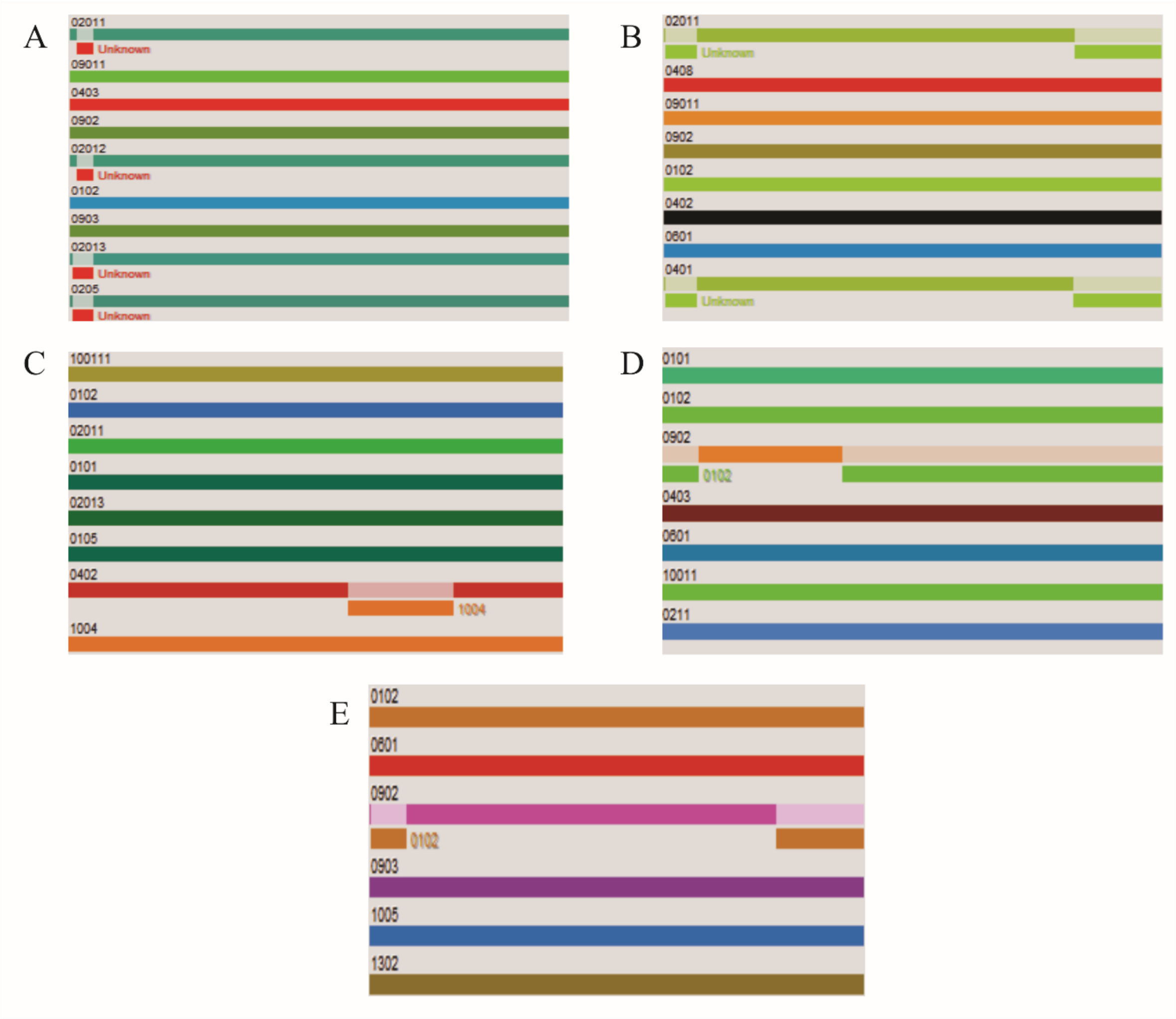

Table S3. Distribution of allele frequency in the population.

Allele	COLOMBIAN POPULATION						WORLDWIDE POPULATION												
	R	M	U	CM	SP	Z	WB	Y	B	D	L	L-C	Y-C	E	KNP	MM	SNU	P	A
*01:01	0.048	0.645	0.200	0	0	0	0.044	0.258	0.048	0	0.050	0	0	0.181	0.200	0	0	0.057	0.016
*01:02	0.161	0.112	0.328	0.066	0.028	0.038	0	0.016	0	0	0.150	0	0	0.086	0	0	0	0	0.188
*01:03	0	0	0	0	0	0	0	0	0	0	0	0	0	0.009	0	0	0	0	0
*01:04	0	0	0	0	0	0	0	0	0	0	0	0	0	0.069	0	0	0	0	0
*01:05	0.016	0	0	0	0	0	0	0	0	0	0	0	0	0	0	0	0	0	0.016
*02:01:01	0.064	0	0.357	0.783	0.471	0.403	0.140	0.096	0.290	0.703	0.066	0	0.233	0.190	0	0	0.219	0	0.141
*02:01:02	0	0	0	0.016	0.057	0	0	0	0	0	0	0	0	0	0	0	0	0	0
*02:01:03	0.032	0	0	0	0.028	0	0	0	0	0	0	0	0	0	0	0	0	0	0
*02:02	0	0	0	0	0	0	0	0	0	0	0	0	0	0.009	0	0	0	0	0
*02:05	0	0	0	0	0.014	0	0	0	0	0	0	0	0	0	0	0	0	0	0
*02:10	0	0	0	0	0	0	0.004	0	0	0	0	0	0	0	0	0	0	0	0
*02:11	0	0.016	0	0	0	0	0.052	0	0	0	0	0	0	0	0	0	0	0	0
*03:01	0	0	0	0	0	0	0	0	0	0	0	0	0	0	0	0	0.412	0	0
*03:02	0	0	0	0	0	0	0	0	0	0	0	0	0	0	0.085	0	0	0	0
*04:01	0	0	0	0	0	0.019	0	0.016	0	0	0	0	0	0	0	0	0.210	0.038	0
*04:02	0.016	0	0	0.033	0	0.038	0	0.145	0.032	0.015	0.066	0	0.100	0	0	0	0	0	0
*04:03	0	0.064	0	0.016	0.085	0	0	0	0	0	0.033	0	0	0	0.066	0.142	0	0.076	0
*04:04	0	0	0	0	0	0	0.068	0.064	0.032	0	0.033	0	0	0	0	0	0.157	0.173	0
*04:08	0	0	0	0	0	0.288	0.004	0	0	0.078	0	0	0	0	0	0	0	0	0
*04:09	0	0	0	0	0	0	0.016	0	0	0	0	0	0	0	0	0	0	0	0
*05:01	0	0	0	0	0	0	0	0	0	0.093	0	0.045	0	0	0	0	0	0.019	0
*05:02	0	0	0	0	0	0	0.004	0	0	0	0	0	0	0	0	0	0	0	0

***06:01**	0	0.048	0.057	0.083	0	0.019	0	0	0	0	0	0.272	0	0	0	0.035	0	0	0
***06:02**	0	0	0	0	0	0	0.048	0	0	0	0	0.159	0	0	0	0	0	0.134	0
***06:03**	0	0	0	0	0	0	0	0.064	0.145	0.015	0.2	0	0	0	0	0	0	0	0
***06:07**	0	0	0	0	0	0	0.016	0	0	0	0	0	0.033	0	0	0	0	0	0
***07:01**	0	0	0	0	0	0	0.016	0.096	0	0	0	0	0.033	0.121	0	0.107	0	0.019	0.234
***07:04**	0	0	0	0	0	0	0	0	0	0	0	0	0	0	0	0	0	0	0.047
***08:01**	0	0	0	0	0	0	0.152	0	0	0	0	0.113	0	0	0	0.142	0	0	0
***09:01:01**	0	0	0	0	0.200	0.134	0.036	0.032	0.370	0.093	0.133	0	0.133	0.103	0.004	0.107	0	0.269	0.281
***09:02**	0	0.096	0.014	0	0.085	0.057	0	0	0	0	0	0	0	0	0	0	0	0	0
***09:03**	0	0	0.014	0	0.028	0	0	0	0	0	0	0	0	0	0	0	0	0	0
***09:05**	0	0	0	0	0	0	0	0	0	0	0	0	0	0.009	0	0	0	0	0
***09:06**	0	0	0	0	0	0	0	0	0	0	0	0	0	0.009	0	0	0	0	0
***09:07**	0	0	0	0	0	0	0	0	0	0	0	0	0	0.009	0	0	0	0	0
***10:01:01**	0.645	0.016	0	0	0	0	0.128	0.112	0.016	0	0.266	0.409	0.466	0.164	0.342	0.285	0	0.096	0.047
***10:01:02**	0	0	0	0	0	0	0.004	0	0	0	0	0	0	0	0	0	0	0	0
***10:04**	0.016	0	0	0	0	0	0	0	0	0	0	0	0	0	0	0	0	0	0
***10:05**	0	0	0.014	0	0	0	0	0	0	0	0	0	0	0	0	0	0	0	0.016
***10:06**	0	0	0	0	0	0	0	0	0	0	0	0	0	0	0	0	0	0	0.016
***10:07**	0	0	0	0	0	0	0	0	0	0	0	0	0	0.009	0	0	0	0	0
***10:08**	0	0	0	0	0	0	0	0	0	0	0	0	0	0.009	0	0	0	0	0
***11:01**	0	0	0	0	0	0	0	0	0	0	0	0	0	0	0.3	0	0	0	0
***11:02**	0	0	0	0	0	0	0	0.080	0	0	0	0	0	0	0	0	0	0	0
***11:03**	0	0	0	0	0	0	0	0	0	0	0	0	0	0	0	0.071	0	0	0
***11:09**	0	0	0	0	0	0	0.008	0	0	0	0	0	0	0	0	0	0	0	0
***12:05**	0	0	0	0	0	0	0.016	0	0	0	0	0	0	0	0	0	0	0	0
***13:01**	0	0	0	0	0	0	0.036	0.016	0.064	0	0	0	0	0.069	0	0	0	0.115	0
***13:02**	0	0	0.014	0	0	0	0	0	0	0	0	0	0	0.009	0	0	0	0	0

***14:01**	0	0	0	0	0	0	0	0	0	0	0	0	0	0.009	0	0.107	0	0	0
***14:03**	0	0	0	0	0	0	0.208	0	0	0	0	0	0	0	0	0	0	0	0

R (Remanso), M (Manta), U (Ubaque), CM (Casco Mula), SP (San Pedreño), Z (Zungo), WB (Wild Boar), and (Yorkshire), B (Berkshire), D (Duroc), L (Landrace), L_C (Landrace Europe), Y_C (Yorkshire Europe), E (Europe), KNP (Korean Native Pigs), MM (Microminipig), SNU (Seoul National University), P (Pietrain) and A (Asia).

Table S4. AMOVA results.

Source of variation	d.f.	Sum of squares	Variance components	Percentage of variation
Among populations	16	3264.119	2.32670	17.37
Within populations	1449	16036.607	11.06736	82.63
Total	1465	19300.726	13.39406	

Fixation Index FST : 0.17371

P-value = 0.00000

Fig S5. Progression of populations assignment.

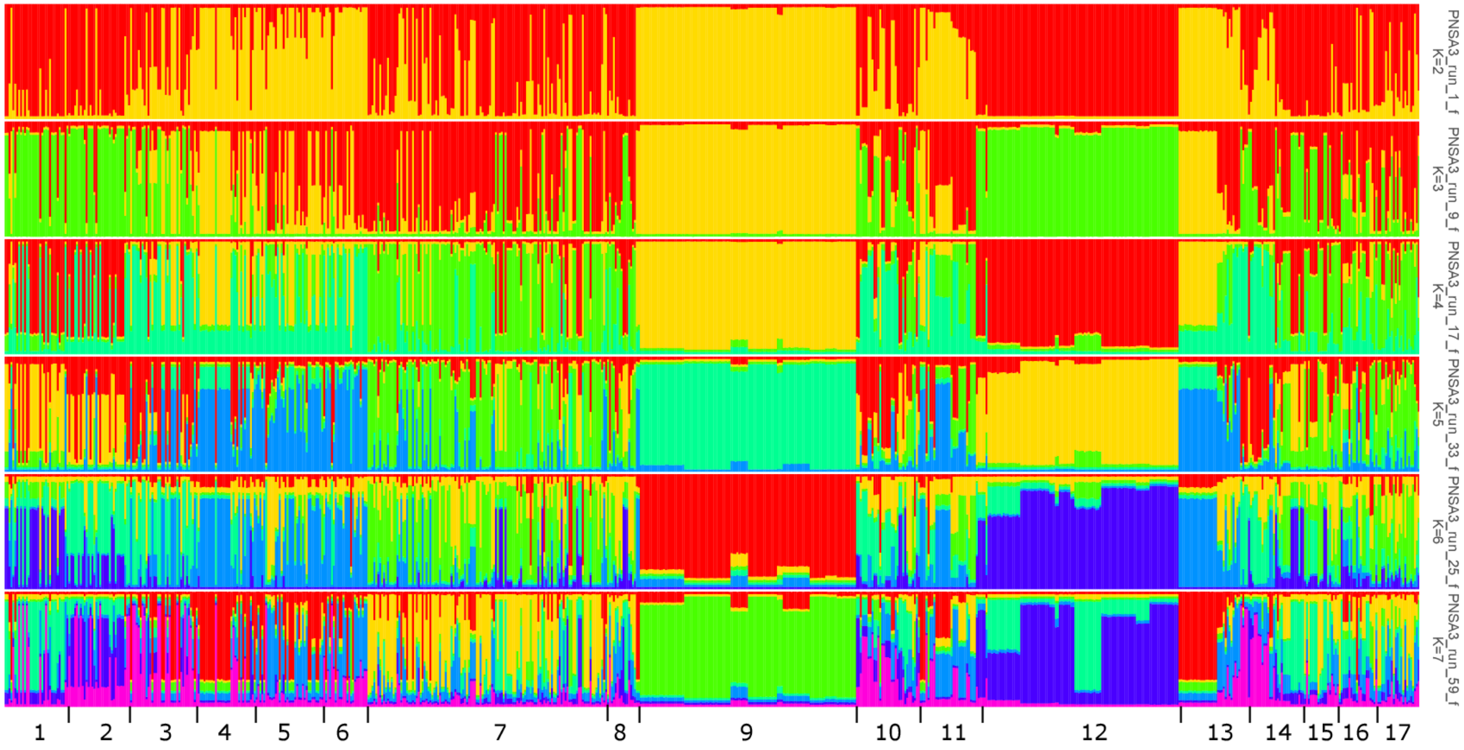

Numbers under (b) indicates populations: 1, Remanso; 2, Manta; 3, Ubaque; 4, Casco Mula; 5, San Pedreño; 6, Zungo; 7, Wild boar; 8, Micromini; 9, SNU; 10, Yorkshire; 11, Berkshire; 12, KNP; 13, Duroc; 14, Landrace; 15, Landrace_C; 16, Yorkshire_C; 17, Pietrain.

Table S6. Complete genotypes of the population. a) Commercial line population and b) Creole pigs population.

a)

Commercial Line								
REMANSO			MANTA			UBAQUE		
ID	ALLELE 1	ALLELE 2	ID	ALLELE 1	ALLELE 2	ID	ALLELE 1	ALLELE 2
SLA-1	*10:01:01	*10:01:01	SLA-32	*09:02	*09:02	SLA-92	*02:01:01	*06:01
SLA-2	*10:01:01	*10:01:01	SLA-33	*01:01	*01:01	SLA-93	*01:02	*06:01
SLA-3	*02:01:01	*10:01:01	SLA-35	*01:01	*06:01	SLA-94	*01:02	*01:01
SLA-4	*02:01:01	*02:01:01	SLA-38	*01:01	*01:01	SLA-95	*02:01:01	*02:01:01
SLA-5	*01:02	*10:01:01	SLA-39	*01:01	*01:01	SLA-97	*01:02	*01:02
SLA-6	*02:01:01	*10:01:01	SLA-41	*01:02	*01:01	SLA-98	*01:02	*01:02
SLA-7	*10:01:01	*10:01:01	SLA-44	*01:02	*01:02	SLA-100	*01:02	*10:05
SLA-8	*01:02	*01:02	SLA-45	*01:01	*01:01	SLA-101	*02:01:01	*01:02
SLA-9	*10:01:01	*10:01:01	SLA-49	*01:02	*01:01	SLA-102	*02:01:01	*01:01
SLA-10	*01:02	*01:02	SLA-50	*01:01	*01:01	SLA-103	*01:02	*01:02
SLA-11	*10:01:01	*10:01:01	SLA-51	*04:03	*04:03	SLA-104	*01:02	*01:01
SLA-12	*01:02	*01:02	SLA-52	*09:02	*09:02	SLA-105	*02:01:01	*09:03
SLA-13	*01:02	*10:01:01	SLA-53	*01:01	*01:01	SLA-106	*01:01	*01:01
SLA-14	*10:01:01	*10:01:01	SLA-54	*01:02	*01:01	SLA-107	*02:01:01	*01:01
SLA-15	*10:01:01	*10:01:01	SLA-55	*01:01	*01:01	SLA-108	*02:01:01	*02:01:01
SLA-16	*10:01:01	*10:01:01	SLA-58	*02:01:01	*01:01	SLA-109	*02:01:01	*01:01
SLA-17	*10:01:01	*10:01:01	SLA-60	*09:02	*09:02	SLA-110	*02:01:01	*01:01
SLA-18	*10:01:01	*10:01:01	SLA-61	*01:01	*01:01	SLA-111	*01:02	*01:02
SLA-19	*04:02	*10:04	SLA-62	*01:02	*01:02	SLA-112	*01:02	*01:02
SLA-20	*10:01:01	*10:01:01	SLA-64	*01:01	*01:01	SLA-115	*02:01:01	*02:01:01
SLA-21	*01:01	*10:01:01	SLA-65	*01:01	*01:01	SLA-116	*02:01:01	*02:01:01
SLA-22	*01:02	*01:02	SLA-70	*01:01	*01:01	SLA-119	*01:02	*06:01
SLA-23	*01:01	*10:01:01	SLA-74	*01:01	*01:01	SLA-120	*02:01:01	*01:01
SLA-24	*02:01:03	*02:01:03	SLA-79	*01:01	*06:01	SLA-121	*01:02	*06:01
SLA-25	*10:01:01	*10:01:01	SLA-82	*10:01:01	*01:01	SLA-122	*02:01:01	*02:01:01
SLA-26	*10:01:01	*10:01:01	SLA-83	*04:03	*04:03	SLA-123	*02:01:01	*02:01:01
SLA-27	*10:01:01	*10:01:01	SLA-85	*01:01	*06:01	SLA-124	*02:01:01	*02:01:01
SLA-28	*01:05	*10:01:01	SLA-86	*01:01	*01:01	SLA-125	*01:02	*01:02
SLA-29	*02:01:01	*01:01	SLA-87	*01:01	*01:01	SLA-126	*02:01:01	*02:01:01
SLA-30	*10:01:01	*10:01:01	SLA-89	*01:01	*01:01	SLA-127	*13:02	*01:01
SLA-31	*10:01:01	*10:01:01	SLA-90	*01:01	*01:01	SLA-128	*01:01	*01:01
						SLA-129	*01:02	*01:02
						SLA-130	*09:02	*01:01
						SLA-131	*01:02	*01:01
						SLA-132	*01:02	*02:01:01

b)

CREOLE PIGS								
San Pedreño			Casco Mula			Zungo		
ID	ALLELE 1	ALLELE 2	ID	ALLELE 1	ALLELE 2	ID	ALLELE 1	ALLELE 2
SLA-1C	*04:03	*04:03	SLA-35C	*02:01:01	*06:01	SLA-67C	*02:01:01	*02:01:01
SLA-2C	*04:03	*02:01:01	SLA-36C	*02:01:01	*02:01:01	SLA-68C	*09:01:01	*02:01:01
SLA-3C	*02:01:01	*02:01:01	SLA-37C	*02:01:01	*01:02	SLA-69C	*09:01:01	*09:01:01
SLA-4C	*02:01:01	*09:01:01	SLA-38C	*02:01:01	*04:03	SLA-70C	*04:02	*02:01:01
SLA-5C	*02:01:01	*09:02	SLA-40C	*02:01:01	*02:01:01	SLA-71C	*02:01:01	*02:01:01
SLA-7C	*02:01:01	*02:01:01	SLA-41C	*02:01:01	*02:01:01	SLA-72C	*04:08	*04:08
SLA-8C	*02:01:01	*04:03	SLA-42C	*02:01:01	*02:01:01	SLA-73C	*02:01:01	*09:01:01
SLA-9C	*02:01:01	*02:01:01	SLA-43C	*02:01:01	*02:01:01	SLA-74C	*01:02	*01:02
SLA-10C	*01:02	*01:02	SLA-44C	*02:01:01	*02:01:01	SLA-76C	*02:01:01	*06:01
SLA-11C	*02:05	*02:01:02	SLA-45C	*02:01:01	*02:01:01	SLA-77C	*02:01:01	*02:01:01
SLA-12C	*02:01:02	*09:03	SLA-46C	*02:01:01	*02:01:01	SLA-78C	*04:08	*04:08
SLA-13C	*09:03	*09:01:01	SLA-47C	*02:01:01	*02:01:01	SLA-79C	*02:01:01	*09:01:01
SLA-14C	*02:01:02	*09:01:01	SLA-49C	*01:02	*01:02	SLA-80C	*02:01:01	*09:01:01
SLA-15C	*02:01:01	*04:03	SLA-50C	*02:01:01	*02:01:01	SLA-81C	*09012	*04:08
SLA-16C	*09:02	*09:02	SLA-51C	*02:01:01	*02:01:01	SLA-82C	*02:01:01	*04:08
SLA-17C	*02:01:01	*02:01:01	SLA-52C	*02:01:01	*02:01:01	SLA-85C	*02:01:01	*02:01:01
SLA-18C	*02:01:02	*09:02	SLA-53C	*02:01:01	*02:01:01	SLA-86C	*02:01:01	*04:08
SLA-19C	*04:03	*02:01:01	SLA-54C	*02:01:01	*02:01:01	SLA-87C	*02:01:01	*04:01
SLA-20C	*02:01:01	*02:01:01	SLA-55C	*02:01:01	*02:01:01	SLA-88C	*02:01:01	*02:01:01
SLA-21C	*09:01:01	*09:01:01	SLA-56C	*02:01:01	*02:01:01	SLA-89C	*09:02	*09:02
SLA-22C	*02:01:01	*02:01:01	SLA-57C	*02:01:01	*06:01	SLA-90C	*09:01:01	*04:08
SLA-23C	*02:01:01	*02:01:01	SLA-58C	*02:01:01	*01:02	SLA-91C	*02:01:01	*04:08
SLA-24C	*02:01:01	*02:01:01	SLA-59C	*04:02	*02:01:01	SLA-92C	*04:08	*04:02
SLA-25C	*09:01:01	*09:01:01	SLA-60C	*06:01	*06:01	SLA-93C	*02:01:01	*09:02
SLA-26C	*02:01:01	*09:02	SLA-61C	*02:01:01	*02:01:01	SLA-96C	*04:08	*04:08
SLA-27C	*02:01:01	*09:01:01	SLA-62C	*02:01:02	*06:01	SLA-97C	*04:08	*04:08
SLA-28C	*09:01:01	*09:01:01	SLA-63C	*02:01:01	*02:01:01			
SLA-29C	*02:01:01	*09:02	SLA-64C	*04:02	*02:01:01			
SLA-30C	*09:01:01	*09:01:01	SLA-65C	*02:01:01	*02:01:01			
SLA-31C	*02:01:03	*02:01:03	SLA-66C	*02:01:01	*02:01:01			
SLA-32C	*02:01:01	*02:01:01						
SLA-33C	*02:01:01	*02:01:01						
SLA-34C	*09:01:01	*02:01:01						
SLA-94C	*02:01:01	*09:01:01						
SLA-98C	*02:01:01	*02:01:01						
